# The Role of ICP Monitoring in Minimally Invasive Surgery for the Management of Intracerebral Hemorrhage

**DOI:** 10.1007/s12975-023-01219-4

**Published:** 2023-12-29

**Authors:** Ge Zhang, Yunjie Li, Danyang Chen, Zhuojin Wu, Chao Pan, Ping Zhang, Xingwei Zhao, Bo Tao, Han Ding, Cai Meng, Diansheng Chen, Wenjie Liu, Zhouping Tang

**Affiliations:** 1 Department of Neurology, Tongji Hospital, Tongji Medical College, Huazhong University of Science and Technology, Wuhan, 430030 China; 2 State Key Laboratory of Digital Manufacturing Equipment and Technology, School of Mechanical Science and Engineering, Huazhong University of Science and Technology, Wuhan, 430074 Hubei China; 3 School of Mechanical Engineering and Automation, Beihang University, Beijing, 100191 China; 4 Beijing WanTeFu Medical Apparatus Co., Ltd., Beijing, China

**Keywords:** Intracranial pressure, Monitoring, Intracerebral hemorrhage, Minimally invasive surgery

## Abstract

Intracerebral hemorrhage (ICH) is the second major stroke type, with high incidence, high disability rate, and high mortality. At present, there is no effective and reliable treatment for ICH. As a result, most patients have a poor prognosis. Minimally invasive surgery (MIS) is the fastest treatment method to remove hematoma, which is characterized by less trauma and easy operation. Some studies have confirmed the safety of MIS, but there are still no reports showing that it can significantly improve the functional outcome of ICH patients. Intracranial pressure (ICP) monitoring is considered to be an important part of successful treatment in traumatic brain diseases. By monitoring ICP in real time, keeping stable ICP could help patients with craniocerebral injury get a good prognosis. In the course of MIS treatment of ICH patients, keeping ICP stable may also promote patient recovery. In this review, we will take ICP monitoring as the starting point for an in-depth discussion.

## Introduction

Intracerebral hemorrhage is the hemorrhage of blood in the cerebral parenchyma, accounting for 27.9% of all new strokes. The annual incidence is 36.53~47.88/100,000 people, and the case-fatality rate is 32.98~38.67/100,000 people. It causes a heavy burden of disease for the society and families [1]. The most critical factors affecting the prognosis of ICH patients are hematoma volume and location. Secondary injuries such as inflammatory response are also strongly associated with prognosis [2, 3]. At present, the treatment of ICH includes medical treatment and surgical treatment. Surgery has become an important method for the treatment of ICH because of its advantages including rapid hematoma removal, intracranial hypertension relief, mechanical compression relief, and alleviation of neurotoxicity and inflammatory cascade reactions. Surgery mainly includes craniotomy and MIS (methods including catheters and machinery). Accurate selection of patients with surgical indications for ICH treatment is crucial to treatment success [4]. In the 2022 AHA/ASA guidelines for the management of patients with spontaneous ICH, MIS is recommended for the removal of a hematoma associated with supratentorial ICH and intraventricular hemorrhage (IVH) as it has a lower incidence of mortality than drug therapy alone. None of the clinical studies in previous years have confirmed that surgical treatment significantly improves the prognosis of ICH patients [5]. However, there is now a turnaround in this predicament. The early minimally invasive removal of intracerebral hemorrhage (ENRICH) trial is a randomized controlled clinical trial evaluating the efficacy of a minimally invasive transcallosal fascial pars operas (MIPS) approach for the treatment of supratentorial ICH [6]. The results of the ENRICH trial were made public for the first time at the 2023 American Association of Neurological Surgeons (ANNS) Conference. The results show that surgical treatment significantly improves the 6-month functional prognosis, reduces 30-day mortality, shortens the length of ICH hospitalization, and lowers costs of ICH patients. We await details as to the results of this trial. In addition, data from a recent systematic review showed that endoscopic treatment of IVH patients reduced the length of ICH hospitalization and the risk of persistent cerebrospinal fluid diversion [7].

The results of the minimally invasive surgery plus alteplase for intracerebral hemorrhage evacuation (MISTIE III) trial indicated that to successfully evacuate a hematoma, its volume must be reduced by more than 70% or its residual volume must be less than 15 mL to achieve good functional outcomes at 1 year, thereby proving that the success of evacuation is related to functional outcomes [8]. The success of hematoma removal is first related to the experience of the surgeon. Second, optimizing the surgical route can reduce the incidence of sustaining an iatrogenic injury, thus facilitating the complete removal of the hematoma. Finally, the development of a hematoma removal strategy needs to balance the need to achieve a good hemostatic effect with its occupying effect on its pulling of the surrounding tissue and toxicity. ICP was increased by the intracranial mass effect, hematoma expansion, cerebral edema, and changes in cerebrospinal fluid circulation dynamics after ICH treatment, which decreased following the removal of the hematoma. The average incidence of increased ICP after cerebral hemorrhage is about 67% [9]. MIS reduces elevated ICP and reduced CPP duration after ICH, which is significantly associated with improved patient prognosis [10]. However, it is important to note that surgery may also lead to a rapid decrease in intracranial pressure, which in turn may cause rebleeding and cerebral edema. Typically, ICP decreased slowly during MIS, whereas ICP decreased rapidly in the major craniotomy group. After completion of hematoma removal, ICP decreased to normal levels in equal proportions in both groups [11]. Previous studies have shown that postoperative ICP monitoring is significantly associated with 3-day mortality after ICH treatment and the Glasgow Outcome Score (GOS) at discharge. ICP monitoring can provide real-time feedback on the local intracranial environment and help to lower intracranial pressure [12, 13].

Based on the above discussion, MIS has certain advantages in hematoma removal. Early hematoma removal, reduced incidence of iatrogenic injury and prevention of rebleeding are the goals of ICH treatment [14]. To further improve the functional outcomes of patients undergoing MIS for ICH and make MIS the most promising surgical strategy for ICH patients, Feng Hua proposed exploring the spatial relationship between the hematoma and corticospinal tract during MIS to reduce iatrogenic injury [15–17]. In this paper, we propose the idea of guiding MIS through intraoperative intracranial pressure monitoring, thereby balancing the advantages of hematoma compression and hemostasis and reducing the incidence of damage of the surrounding brain tissue caused by hematoma through stable reduction of intracranial pressure, optimizing the hematoma removal strategy and improving the removal efficiency to ultimately improve the functional outcomes of ICH patients.

## Search Methods and Study Eligibility

An electronic search of studies published up to May 5, 2023 was conducted on PubMed in accordance with the PRISMA (Preferred Reporting Items for Systematic Review and Meta-analysis) guidelines [18], using the MeSH topic words “Intracranial Pressure,” “Intracerebral Hemorrhage,” and “Hemorrhagic Stroke.” The objective was to focus on the pathophysiology of ICP changed after ICH, clinical trial, and significance of ICP monitoring in ICH, and the selection of appropriate monitoring methods. Studies that fell outside the scope and intent of this review were excluded.

**Fig. 1:**
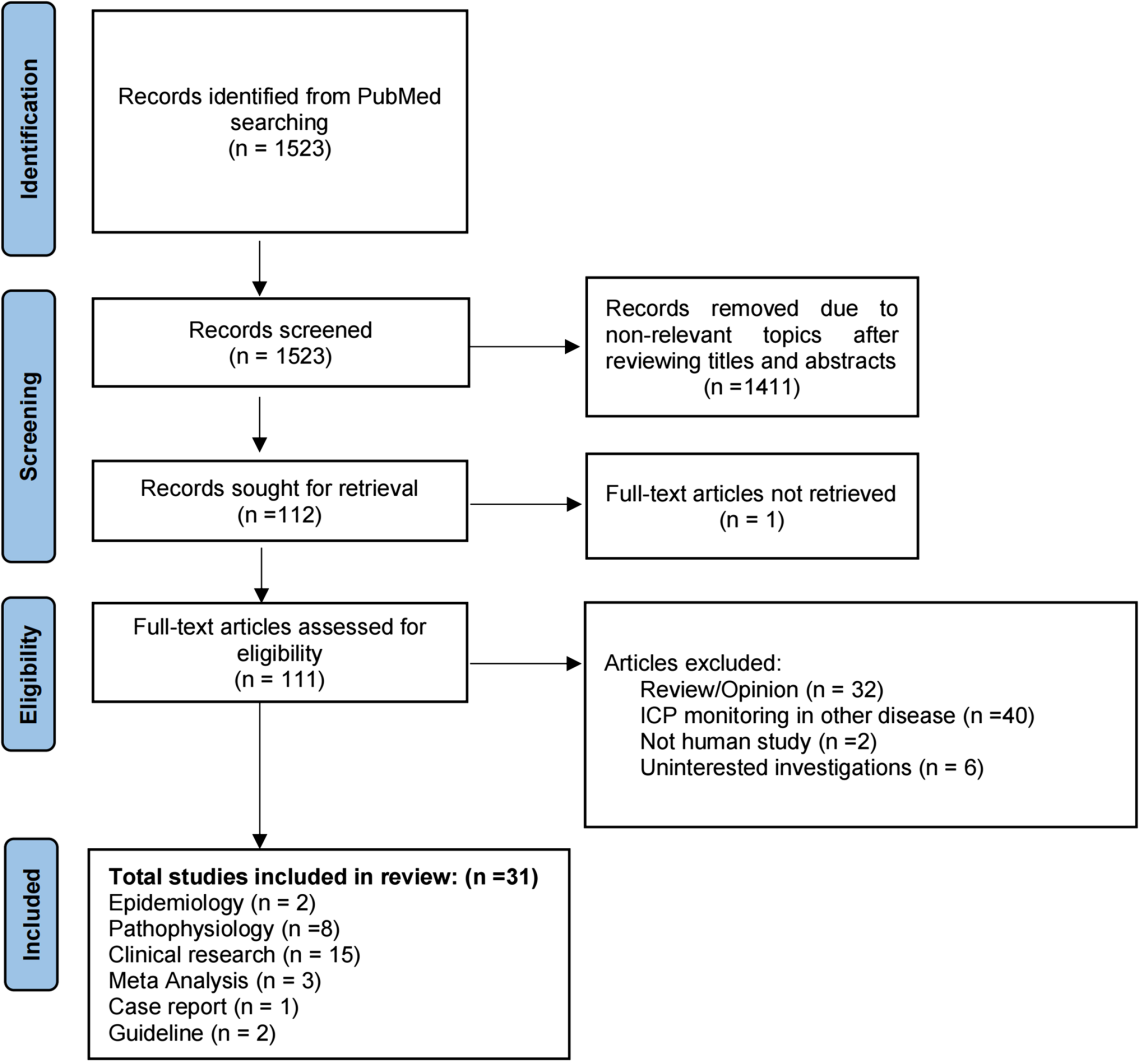
Flow diagram of study identification and selection. PRISMA study flow diagram demonstrating the number of articles retained at each stage of data acquisition

The first author assessed all relevant studies and the relevant references in each article. Risk of bias was not assessed because of significant variability in the studies included in this review. The search results were presented in Fig. 1.

## Theoretical Basis of ICP Monitoring After ICH

After the occurrence of cerebral hemorrhage, the space-occupying effect of hematoma and the edema of perihematoma increases the intracranial volume, and the ICP increases according to the law of intracranial volume- pressure curve after exceeding the co-compensatory regulation effect of cerebrospinal fluid and cerebral blood flow [5, 19–22].

Previous studies have shown that elevated ICP is easily associated with ICH. Godoy et al. analyzed six clinical trials and found that the incidence of an elevated ICP (ICP > 20 mmHg) after the development of an ICH was 67% (95% confidence interval (*CI*) 51~84%) [9]. In the MISTIE III trial, 43.1% of ICH patients had an ICP ≥ 20 mmHg, and 16.7% had an ICP ≥ 30 mmHg. In total, 72.2% of ICH patients had a cerebral perfusion pressure (CPP) ≤ 70 mmHg, and 34.7% of patients had a CPP ≤ 60 mmHg [8, 10]. Among ICH patients with obstructive IVH who were enrolled in the clot lysis: evaluating accelerated resolution of intraventricular hemorrhage (CLEAR III) trial, 73% had an elevated ICP [23]. After an ICH developed, the increase in ICP was mainly caused by enlargement of the intracranial hematoma, resulting in focal elevated ICP [24]. This causes a pressure gradient between the two hemispheres of the brain, which can lead to line migration and, in severe cases, herniation [25]. The brain needs constant CBF to maintain normal metabolic activity. CBF is related to CPP and cerebral vascular resistance (CVR), namely, CBF = CPP/CVR. A CPP is equal to the mean arterial pressure (MAP) minus the ICP. Therefore, when the ICP changes, the cerebral vascular regulation function maintains a stable CBF by regulating cerebral artery resistance. Hypoperfusion and cerebral edema may occur when the CPP’s automatic regulation range (50~100 mmHg) is exceeded [21]. After an ICH occurs, the effects of the damage caused by compression of the hematoma on the peripheral cerebral vessels decrease the local CBF, leading to peripheral cerebral tissue ischemia, hypoxia, and cerebral edema [26]. The occurrence of cerebral edema further increases an elevated ICP, decreases the CPP, and decreases the CBF, forming a vicious cycle and aggravating a brain injury [27]. A decreased CPP was also associated with the onset of a new ischemic stroke (IS) within 30 days after ICH development [23]. These results indicate that ICP plays an important role in injury after ICH.

After ICH, a pressure gradient is generated between the hematoma and the brain tissue, which promotes the displacement of the brain tissue, and at the same time, the ischemia and hypoxia of the brain tissue around the hematoma aggravate the cerebral edema. A sharp rise in ICP can cause brain herniation in severe cases. Toxic factors are produced during hematoma dissipation, further damaging the surrounding brain tissue, causing cerebral edema and exacerbating ICP elevation [28, 29]. In addition, cerebral hypoperfusion after ICH may be due to elevated ICP. However, it has been shown that ICH patients who have normal ICP may also experience hypoperfusion, which may be a state of congestion [30, 31]. During the removal of the hematoma, the pressure in the hematoma cavity decreases continuously, creating a negative pressure gradient with the surrounding brain tissue, which promotes the reduction of the brain tissue. Hematoma removal can be divided into passive removal and active removal according to whether suction pressure is applied. Passive removal involves thrombolysis and passive catheter drainage using a YL-1 puncture needle combined with urokinase. Active clearance involves emptying the hematoma during the operation without indwelling the drainage tube or performing thrombolysis [32]. Sun et al. studied the changes in ICP in different surgical procedures. In patients with craniotomy, ICP falls rapidly after craniotomy and slowly and unevenly during hematoma resorption. This causes traction on the surrounding brain tissue thereby damaging the brain tissue and affecting its homing. With endoscopic keyhole, ICP does not drop rapidly after craniotomy due to the small bone aperture. After the formation of a cortical fistula, the cerebral hematoma and the surrounding tissue form a reverse pressure gradient, which promotes the surrounding deviated brain tissue to return to its place, and the cerebral hematoma actively enters the tube. The endoscopic tube is columnar in shape, which creates uniform pressure on the surrounding brain tissue and avoids unnecessary traction injury [11]. Stereotactic aspiration and thrombolytic techniques used in the MISTIE III trial were similar to keyhole endoscopy in that intraoperative ICP decreased slowly with hematoma clearance. Al-Kawaz et al. found in the MISTIE III trial that reducing the hematoma volume through MIS combined with rt-PA could reduce the proportion of high ICP and decrease CPP events [10]. However, when the residual cavity is large, there is a risk that the cavity may collapse and close due to incorrect aspiration, and the residual hematoma is isolated in a separate cavity, which is not conducive to the complete removal of the hematoma [33, 34]. Therefore, it is particularly important to control and monitor intraoperative suction negative pressure and ICP. A constant and smooth pressure difference between the intracranial and hematoma cavities during the surgery procedure is more conducive to rapid and complete removal of the hematoma, which is most beneficial to the protection of the surrounding brain tissue (reduction of cerebral edema and restoration of cerebral blood flow). It is worth noting that combined monitoring of cerebral oxygenation pressure along with monitoring of ICP during MIS improves the accuracy of perivascular blood flow perfusion [35]. Reducing ICP stably and removing the hematoma to the greatest extent is the top priority of surgical treatment for ICH patients.

## Clinical Relevance

### Guideline Recommendation

Studies have shown that patients with an elevated ICP after ICH treatment have higher mortality [9]. In the 2022 AHA/ASA Guidelines for the management of patients with spontaneous ICH, ICP monitoring and treatment are suggested to help reduce the incidence of mortality and to improve outcomes in patients with moderate-severe spontaneous ICH with a decreased level of consciousness (Glasgow Coma Scale (GCS) ≤ 8) (evidence level IIb). ICP monitoring is recommended for patients with a GCS score of 3~8 and an ICP maintained at < 22 mmHg, and a CPP maintained at 50~70 mmHg are needed for brain autoregulation [5]. The European Stroke Organization does not recommend ICP monitoring due to a lack of RCT trials [36]. The role of ICP monitoring in traumatic brain injury (TBI) has been widely studied and accepted [22]. However, research on ICH is limited. Therefore, this study focuses on the changes in ICP after ICH treatment, emphasizes the importance of ICP monitoring in the clinical management of ICH patients, and proposes suggestions for individualized treatment based on ICP.

### Selection of Intracranial Pressure Monitoring Methods

ICP monitoring methods can be classified as invasive and non-invasive. Intraventricular pressure (IVP) monitoring is regarded as the gold standard for ICP monitoring and can be used for CSF drainage and treatment as well as whole-brain ICP monitoring. However, this method is not suitable for patients with compression or displacement of the ventricle who cannot be continuously monitored. Some studies suggest that for ICH patients, there is a pressure difference between near and far hematomas, and external ventricular drainage (EVD) may not accurately represent the local space-occupying effect caused by the hematoma and perifocal edema [37, 38]. In recent years, the monitoring of brain parenchyma pressure (BPP) using optical fiber sensors has been developed and popularized in clinical practice [39]. BPP is suitable for continuous monitoring and is suitable for the whole brain or local ICP. The measurement error of BPP is mainly from the zero drift. Microtransducers are often placed in the right frontal cortex and parenchyma but can be placed in other locations as needed. Its advantages include simple readout, independence from patient position, easy surgical maneuvering, low incidence of bleeding and infection, and few measurement artifacts. Its disadvantages include the tendency of the system to drift with prolonged use, the inability to recalibrate, the inability to drain cerebrospinal fluid, and the inability to perform whole-brain ICP monitoring.

Current methods of non-invasive ICP monitoring are based on changes associated with elevated ICP. Evaluation methods, including morphological (assessment by magnetic resonance, computed tomography, transcranial Doppler (TCD), ocular optic sheath diameter ultrasound [40], and fundus microscopy) and physiological (pupil diameter, tympanometry, near-infrared spectroscopy, electroencephalography, visual evoked potential, and otoacoustic emission), were developed [41, 42]. The invasive vs. non-invasive measurement of intracranial pressure in brain injury trial (IMPRESSIT-2) is a prospective multicenter international clinical trial that has identified transcranial Doppler sonography as a screening tool to exclude intracranial hypertension in patients with an acute brain injury (including TBI, ICH, and IS) [43]. In general, non-invasive monitoring is easy to use, inexpensive, accurate, has few contraindications to its use, and is low in infection and bleeding adverse effects compared with invasive monitoring. The potential of non-invasive ICP monitoring to replace the gold standard invasive ICP monitoring has been demonstrated [44]. At present non-invasive ICP monitoring can be used as a screening tool for an elevated ICP, but it is not recommended to guide treatment for ICP control [41, 43, 45–47]. In addition to this, artificial intelligence (AI) can help in finding accurate features to predict elevated ICP from large amounts of mixed and multidimensional data [48]. A recent data for invasive intracranial pressure monitoring in neurocritical care patients suggests that the use of machine learning methods can be effective in predicting intracranial pressure changes in patients in advance. This can provide sufficient response time to alter therapeutic measures [49]. In the future, the trend of medical-industrial crossover will provide more convenient support for intracranial pressure monitoring of cerebral hemorrhage.

In the International Multidisciplinary Multimodal Monitoring consensus meeting, it was suggested that non-TBI patients requiring monitoring should receive an invasive device (parenchymal or intraventricular) rather than a non-invasive device and that intraparenchymal monitors (IPM) and EVD are equally reliable in providing ICP monitoring [50]. Therefore, ICP monitoring has certain advantages in managing ICH post-BPP.

### Clinical Trials Related to ICP Monitoring

Most existing ICP monitoring studies have focused on patients with a TBI, and there are few data on the association between an elevated ICP and neurological deterioration after ICH treatment or whether ICP monitoring helps reduce the incidences of mortality and disability (Table 1). Intracranial pressure monitoring in patients with an acute brain injury in the intensive care unit (SYNAPSE-ICU) is an international prospective observational cohort study analyzing the effect of ICP monitoring on the prognosis of ICH. A weighted Cox regression model showed that ICP monitoring was associated with a significant reduction in 6-month mortality rates (hazard ratio (*HR*) 0.49; 95% *CI* 0.35~0.71; *P* = 0.001) but was not related to neurological outcomes (odds ratio (*OR*) 0.83; 95% *CI* 0.41~1.68; *P* = 0.6077) [51, 52]. The CLEAR III trial conclusions support the combination of ICP and CPP management in ICH treatment, especially for patients with obstructive IVH requiring EVD [23]. The results of the ethnic/racial variations of intracerebral hemorrhage (ERICH) study showed that ICH patients without an IVH who were monitored by ICP had a lower 90-day mortality rate than those who were not monitored by ICP (23.4% vs. 36%; *OR* 0.543; 95% *CI* 0.302~0.975; *P* = 0.041), but there was no improvement in 90-day functional outcomes (modified Rankin score (mRS) 0~2) in these patients (14.4% vs. 28.8%; *OR* 0.416; 95% *CI* 0.203~0.813; *P* = 0.010), and ICP monitoring was associated with increased use of hypertonia therapy, increased surgery rate, and longer hospital stay. This study did not provide evidence to support the routine use of ICP monitoring in ICH patients [38]. Menacho et al.’s secondary analysis of the MISTIE III trial showed that ICP-monitored patients had a higher incidence of new or worsening ICH than unmonitored patients (41.4% vs. 22.9%; *P* = 0.001) and a higher likelihood of intracranial infection (7.1% vs. 1.7%; *P* = 0.006). The infection rate was higher in EVD patients (10.2% vs. 1.7%; *P* < 0.001), and IPM patients had a higher incidence of new or worsening ICH (57.1% vs. 22.9%; *P* < 0.001). The results may be associated with higher disease severity in patients receiving ICP monitoring [53]. In addition, several prospective/retrospective single-center clinical studies with small samples have shown that MIS combined with ICP monitoring is associated with better outcomes in ICH patients [54–57].

**Table 1:** Clinical trials of ICP monitoring in ICH treatment

References	Included patients		Monitoring devices	Outcomes	
	No-ICP	ICP			
SYNAPSE-ICU [52]	306	281	EVD (148, 53.6%) IPM (119, 43.1%) Others (9, 3.3%)	6-month mortality	*HR* = 0.49 95% *CI* (0.35–0.71) *P* = 0.001
				6-month functional outcome	*OR* = 0.83 95% *CI* (0.41–1.68) *P* = 0.6077
ERICH [38]	2434	566	-	90-days mortality	*OR* = 0.543 CI (0.302–0.975) *P* = 0.041
				mRs score 0-2 at 90 days	*OR* = 0.416 CI (0.203–0.813) *P* = 0.010
MISTIE III [53]	424	70	EVD (49, 70%) IPM (21, 30%)	mRs score 4-6 at 1 year	*OR* = 2.37 *CI* (1.15–4.86) *P* = 0.019
				1-year mortality	*OR* = 1.13 CI (0.68–2.60) *P* = 0.411

*ICH* intracerebral hemorrhage, *ICP* intracranial pressure, *SYNAPSE-ICU* intracranial pressure monitoring in patients with acute brain injury in the intensive care unit, *ERICH* ethnic/racial variations of intracerebral hemorrhage, *MISTIE* minimally invasive surgery plus alteplase for intracerebral hemorrhage, *EVD* external ventricular drainage, *IPM* intraparenchymal monitors, *HR* hazard ratio, *OR* odds ratio

Postoperative observation of ICH patients often relies on clinical signs and is then confirmed by imaging methods. Postoperative rebleeding tends to occur in patients with a high ICP due to irritability or pain, but using sedatives may interfere with the assessment of the patient’s consciousness. ICP monitoring can solve this problem by dynamically detecting the patients’ conditions before clinical signs appear and actively guiding clinical intervention [58–60]. Rasulo et al. used brain microdialysis to monitor the metabolic level of the brain tissue around the hematoma (monitoring the contents of glucose, pyruvate, lactic acid, glutamic acid, and glycerol in the injected artificial cerebrospinal fluid) and combined it with patient hemodynamic variables (MAP, ICP, CPP, and pressure reactivity index (PRx)) to predict the prognosis of ICH [61]. Yang et al. proposed that ICP combined with ICP dose (DICP), PRx, and CPP multiparameter ICP monitoring of regression can be used to predict the prognosis of ICH and facilitate individualized treatment of patients [62].

## The Guiding Role of ICP Monitoring in MIS for ICH

### The Significance of ICP Monitoring for MIS in ICH Patients 

The significance of ICP monitoring comes from the guidance of treatment rather than the recording of stress itself [46]. Choosing appropriate surgical methods combined with ICP monitoring to guide postoperative management can effectively control ICP and prevent complications in a timely manner, thus benefitting patients. Because clinical studies such as MISTIE III and ERICH were not used to evaluate the specific results related to ICP monitoring, the type of ICP monitor, the position of the device relative to the hematoma, the duration of monitoring, the ICP waveform, and CPP measurements were not discussed [10, 38]. Studies have shown that the brains of elderly patients have low tolerability [63, 64], poor ability to cope with ICP changes, and greater negative pressure on the wall of the hematoma, which may lead to blood vessel rupture and bleeding. The hemostatic mechanism after ICH is affected not only by the patient’s coagulation function but also by the tamponade effect of the hematoma, which can prevent the expansion of the hematoma [65, 66]. Therefore, it is necessary to prevent rebleeding due to the failure of hemostasis caused by mechanical compression due to an excessively fast or slow reduction in ICP during the operation. The development of intracerebral parenchymal transducers makes it possible to monitor ICP intraoperatively.

Age, GCS score, and initial hematoma volume are all important predictors of ICH functional prognosis [67], but these cannot be changed, and the degree of hematoma clearance and time window of surgical treatment are operable factors [68]. In the MISTIE trials, blood clots were pumped manually through a syringe until resistance was felt, and then a drainage catheter was inserted for thrombolytic medication injection, saline irrigation, and drainage. Hematomas are liquid at the early stage, with strong fluidity and allowing easy suction, but the risk of hematoma dilation and hemostasis needs to be balanced [60]. With the increase in the duration of the disease, blood coagulation, and the increase in fiber components make it difficult to aspirate, and hematoma liquefaction agents (urokinase, rt-PA, etc.) are needed to increase the efficiency of aspiration. Some auxiliary suction devices, such as the NICO BrainPath system and myriad handpiece (NICO Corp., Indianapolis, IN, USA), the Artemis system (Penumbra, Alameda, CA, USA), and The Apollo system (Penumbra Inc., Alameda, CA, USA), can enhance the operator’s control of the suction intensity, improve the efficiency of hematoma removal, and reduce secondary injuries caused by the operation [32, 69–71]. Tang et al. proposed improving the existing minimally invasive hematoma suction and flow, especially in the hematoma suction process. By strictly controlling the initial suction flow and suction negative pressure, the mortality and rebleeding rates of ICH patients who undergo continuous thrombolysis and drainage are lower than those of patients who undergo traditional hematoma suction [72].

### Strategies for Monitoring ICP During MIS in ICH Patients 

Therefore, based on the intracranial volume–pressure curve, we propose smoothing to reduce ICP through real-time monitoring of the ICP of the hematoma cavity, integrating different patient clinical characteristics, such as the hematoma situation (including hematoma location, size, shape, and degree of liquefaction) and the patient characteristics (age, sex, underlying diseases, blood clotting function, arteriovenous malformation, aneurysm, etc.) to individually adjust the suction flow strategy and accurately reduce the intracranial pressure in layers and stages.

**Fig. 2:**
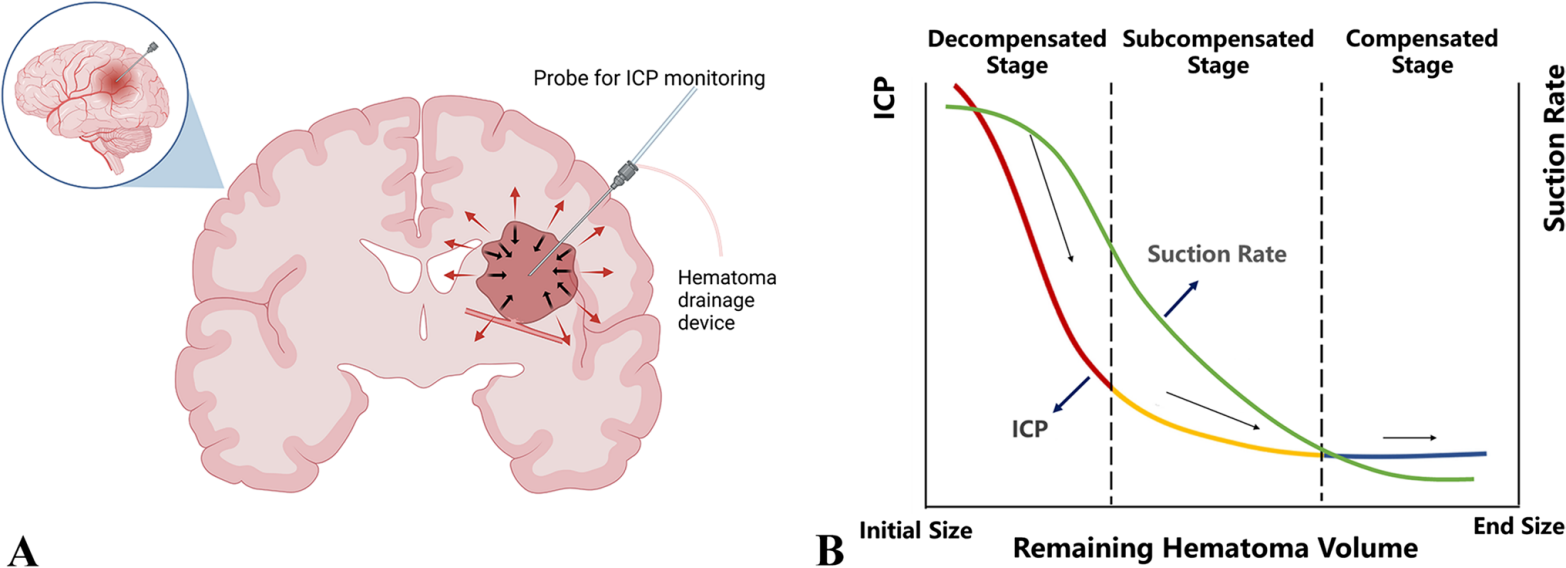
ICP monitoring in the management of intracerebral hemorrhage. **A**. Balance the role of compression hemostasis and the risk of hematoma mass.  **B**. According to the compensatory ability of residual hematoma volume and ICP, the hematoma aspiration strategy was formulated

The key parameters of the suction strategy include the suction rate, suction volume, and use of a liquefaction agent. In the case of a severely elevated ICP, the primary purpose of surgery is to quickly relieve the mass effect of the hematoma, and at this time, large negative pressure can be discharged from the hematoma to rapidly reduce the ICP. When brain compliance gradually recovers, small intracranial volume changes can lead to a sudden drop in the ICP, and at this time, the hematoma cavity generates negative pressure on the surrounding tissues, forming a pulling effect. To prevent a decompression injury and rebleeding, a stable reduction in ICP should be taken as the goal at this stage, and eventually, the residual hematoma should be slowly removed (Fig. 2). To achieve the purpose of stable reduction of ICP, it is necessary to rely on animal experiments and clinical experiments to collect patient data, establish the biological tissue mechanics model of intracranial hematoma, build a big data platform, strengthen learning and constantly optimize the migration to the decision-making model of doctors, and finally realize the generation of intelligent surgical plans. Resources for MIS are limited, and the duration of drainage and extubation depends on multiple postoperative computerized tomography (CT) evaluations of the hematoma. Continuous postoperative ICP monitoring also helps the neurosurgeon be more sensitive to detect any abnormal increase in ICP and initiate timely intervention.

### Prospects of Surgical Robots in MIS and ICP Monitoring of ICH Patients 

Robot-assisted surgery, as a new MIS method for the treatment of ICH, is superior to traditional MIS or drug-conservative treatment in that it has lower rebleeding and intracranial infection rates and has been associated with improved neurological functions [73]. Yan et al. proposed a continuum robot that was designed with a precurved inner tube and a flexible tip for intracranial hematoma suction, which can remove the hematoma more thoroughly and reduce damage to healthy brain tissue [74]. However, in the design of this kind of concentric tube robot, the suction parameters (suction rate, suction volume, etc.) are not discussed in detail [75, 76]. The application of robot-assisted MIS for the treatment of ICH and simultaneous detection of an elevated ICP with multisensor probes is conducive to restoring the intracranial environment after hematoma removal. Furthermore, the characteristics of the patients need to be assessed to allow the creation of a personalized MIS treatment plan for ICH.

## Conclusion

There is still no standardized protocol for post-ICH ICP monitoring. We encourage further research into the underlying mechanisms of an increased ICP and an intracranial compensatory response after ICH treatment to develop more effective treatments. At the same time, whether intraoperative and postoperative management of ICH patients based on ICP monitoring data or fusion multivariate monitoring is effective still needs to be discussed in a large sample prospective cohort clinical study.

## Abbreviations

*ICH*: intracerebral hemorrhage
*MIS*: minimally invasive surgery
*IVH*: intraventricular hemorrhage
*MISTIE III*: minimally invasive surgery plus alteplase for intracerebral hemorrhage
*ICP*: intracranial pressure
*GOS*: Glasgow outcome score
*CSF*: cerebrospinal fluid
*CBF*: cerebral blood flow
*CI*: confidence interval
*CPP*: cerebral perfusion pressure
*CLEAR III*: Clot lysis: evaluating accelerated resolution of intraventricular hemorrhage
*CVR*: cerebral vascular resistance
*MAP*: mean arterial pressure
*IS*: ischemic stroke
*GCS*: Glasgow Coma Scale
*TBI*: traumatic brain injury
*IVP*: intraventricular pressure
*EVD*: external ventricular drainage
*BPP*: brain parenchyma pressure
*IMPRESSIT-2*: invasive vs. noninvasive measurement of intracranial pressure in brain injury trial
*TCD*: transcranial Doppler sonography
*IPM*: intraparenchymal monitors
*SYNAPSE-ICU*: intracranial pressure monitoring in patients with acute brain injury in the intensive care unit
*HR*: hazard ratio
*OR*: odds ratio
*ERICH*: ethnic/racial variations of intracerebral hemorrhage
*mRS*: modified Rankin score
*PRx*: pressure reactivity index
*DICP*: ICP dose
*CT*: computerized tomography
